# Health and voting over the course of adulthood: Evidence from two British birth cohorts

**DOI:** 10.1016/j.ssmph.2019.100531

**Published:** 2019-12-16

**Authors:** Thierry Gagné, Ingrid Schoon, Amanda Sacker

**Affiliations:** a International Centre for Lifecourse Studies in Society and Health, University College London, UK; b Research Department of Epidemiology and Public Health, University College London, UK; c Institute of Education, University College London, UK

**Keywords:** United Kingdom, Voting, Self-rated health, Physical limitations, Life-course, 1958 national child development study, 1970 British cohort study

## Abstract

Systematic differences in voter turnout limit the capacity of public institutions to address the needs of under-represented groups. One critical question relates to the role of health as a mechanism driving these inequalities. This study explores the associations of self-rated health (SRH) and limitations in everyday activities with voting over the course of adulthood in the 1958 National Child Development Study and the 1970 British Cohort Study. We used data from participants who reported voting in the last general election at least once between the ages of 23 and 55 in the 1958 cohort and between the ages of 30 and 42 in the 1970 cohort. We examined associations controlling for a range of early-life and adult circumstances using random-effects models. Compared with those in good or better health: those in fair health had 15% and 18% lower odds of voting in the 1958 and 1970 cohorts; those in poor or worse health had 17% and 32% lower odds of voting in the 1958 and 1970 cohorts. These effects varied with age and were most marked among those in poor health at the ages of 23/30 in the 1958 and 1970 cohorts. Controlling for SRH, having limitations in everyday activities was not associated with voting in main models. Examining age-based differences, however, we found that reporting limitations was associated with a higher probability of voting at the age of 55 in the 1958 cohort and at the age of 30 in the 1970 cohort. Building on the qualities of the British birth cohorts, we offer nuanced evidence about the role of health on voting, which involves considerable life-course processes. Future studies need to examine how these findings progress after the age of 55, extend to mental wellbeing and health practices, and contribute to explain social inequalities in voter turnout.

## Highlights

• Self-rated health (SRH) was associated with a lower probability of voting in adulthood.
• The SRH-voting association was stronger among those born in 1970 compared to 1958.
• The SRH-voting association was stronger in early adulthood compared to midlife.
• Above SRH, reporting limitations in daily activities was not associated with voting.

## 1 Introduction

One key characteristic of modern democratic societies lies in the capacity of its citizens to influence politics through voting in events such as general elections. The legitimacy of this process depends on the equal opportunity to vote across all groups, independent of age, gender, race/ethnicity, family background, and other social characteristics. As a dimension of social capital, the high prevalence and fair distribution of voting is also considered as a determinant of population health (Lantz & Pritchard, 2010). Systematic differences in voter turnout, however, remain relatively common (Smets & van Ham, 2013).

Health-related outcomes such as physical disability and mental illness have been consistently found to predict lower voter turnout, with voting rights in these groups already championed by advocacy groups (Kamens, Blum, & Styron, 2019; Matsubayashi & Ueda, 2014; Schur, Adya & Kruse, 2013; Schur, Shields, Kruse, & Schriner, 2002, Schur, Shields, & Schriner, 2005). The broader role of health in voting has not received the same level of interest yet has been gaining traction over the past five years (Gollust & Rahn, 2015; Mattila, Söderlund, Wass, & Rapeli, 2013). A critical dimension of this debate concerns whether health represents a potential mechanism reinforcing social inequalities in voting over the life-course (Pacheco & Fletcher, 2014; Rodriguez, Geronimus, Bound, & Dorling, 2015). Gollust and Rahn (2015) argued that differences in voting attributable to health may translate into a loss of political power among the socially disadvantaged groups who are less capable to promote their health, thereby representing a new fundamental cause of health inequalities (Phelan, Link, & Tehranifar, 2010).

In this context, this paper seeks to challenge two issues cross-cutting the “health-voting” literature, regarding: 1) the methodological approaches used to assess the robustness of its relationship; 2) the life-course moderators, especially age effects, likely to nuance this relationship over the course of adulthood.

### 1.1 Health and voting: towards a causal association?

Despite newfound interest in the health-voting relationship, public health research on the association between health and voting may be traced back to over twenty years ago (Blakely, Kennedy, & Kawachi, 2001; Smith, 1997; Smith & Dorling, 1996). Studies in the late nineties explored the associations between area-level voter turnout, party affiliation, and mortality rates in the United Kingdom (UK) and found that regions with higher mortality rates were more likely to abstain from voting and vote for left-wing parties (Smith, 1997; Smith & Dorling, 1996). A few years later, Blakely et al. (2001) found that regions in the United States (US) with higher levels of inequalities in voting were more likely to report a high prevalence of poor self-rated health. This work was inspired in part by the rise in popularity of the application of Robert Putnam's social capital theory in public health, which convened that disinvestments in social arrangements worked alongside income inequality to shape population health (Kawachi, Kennedy, Lochner, & Prothrow-Stith, 1997).

Because of the relatively small number of individual-level datasets with data on health and voting, a significant portion of studies in this field is still using aggregate (ecological) designs (Kelleher, Timoney, Friel, & McKeown, 2002, Reitan, 2003; Kim & Kawachi, 2006). For instance, recent studies reported that areas in the US where individuals voted for the Republican Party were more likely to present higher mortality rates and poorer health outcomes (Bilal, Knapp, & Cooper, 2018; Bor, 2017; Wasfy, Stewart, & Bhambhani, 2017). To infer causal relationships from these associations, however, ecological designs build on the critical assumption that associations at the aggregate level are equivalent to associations at the individual level, an argument that has misled experts on the prediction of voter turnout in the past (Gelman, Shor, Bafumi, & Park, 2007; Gnaldi, Tomaselli, & Forcina, 2018).

This first group of studies has been increasingly complemented by individual-level studies. Since the early 2000s, studies on disability and voting have been using large-scale social surveys to examine this relationship (Matsubayashi & Ueda, 2014; Miller & Powell, 2016; Powell & Johnson, 2019; Schur et al., 2002; Schur & Kruse, 2000). Similarly, over the past decade, studies across Canada, the US, and a majority of European countries have found that poor self-rated health is consistently linked to a lower voter turnout (Goerres, 2007; Denny and Doyle, 2007, Denny and Doyle, 2007; Mattila et al., 2013; Pacheco & Fletcher, 2014; Couture & Breux, 2017; Lahtinen, Mattila, Wass, & Martikainen, 2017, Rodriguez, 2018). Some of these studies have also started unpacking the specific conditions (alcoholism, cardiovascular diseases, neurological disorders, co-morbidities) that may inform this association (Gollust & Rahn, 2015; Sund, Lahtinen, Wass, Mattila, & Martikainen, 2017). The vast majority of studies on disability and health, however, have used cross-sectional datasets to assess these associations, precluding us from ruling out issues of temporal ordering and unobserved heterogeneity in most cases.

The most recent wave of studies is tackling these issues. Burden, Fletcher, Herd, Jones, and Moynihan (2016) used a sibling fixed-effects design with American older adults around the age of 70 in the US Wisconsin Longitudinal Survey and found that self-rated health had an equivalent effect on voter turnout to educational attainment at this age. Ojeda and Pacheco (2019) followed the voting behaviour of young adult participants across US national elections up to the age of 29 in the 1997 National Longitudinal Survey of Youth. They found that poor self-rated health at the end of adolescence was associated with a lower probability of voting across elections, but that subsequent changes in self-rated health did not further influence vote behaviour during young adulthood. Similarly, Rapeli, Mattila, &amp; Papageorgiou (2018) examined the repeated association between self-rated health and voting across the 1992, 1997, 2001, and 2005 UK general elections in the British Panel Household Survey (BPHS). They found that the probability of voting among those who rated their health very poorly had been on average six percentage points (p.p.) lower compared to those who rated their health as excellent.

### 1.2 Health and voting: changes over the life-course?

This body of work is consolidating a varied evidence base supporting the robustness of the relationship between health and voting. A second issue, however, concerns the disentanglement of the magnitude of this relationship and its underlying mechanisms at different stages of the life-course. A large literature has established the importance of age as one of the most important predictors of voting (Goerres, 2007; Plutzer, 2002; Smets & van Ham, 2013; Strate, Parrish, Elder, & Ford, 1989). In the United Kingdom, those who are aged 65 + have been on average 32% more likely to vote in a general election compared with those who are aged 18–24 (Dempsey & Loft, 2017). Strate et al. (1989) argued that approximately 50% of this association was explained by increases in knowledge and interest, party affiliation, family income, and social networks over the life-course. Goerres (2007) argued that non-political factors such as stability in residence and marital status also contributed to explain this association.

Few studies, however, have distinguished age-based differences in regard to health and voting. Mattila et al. (2013) examined the age-graded association between self-rated health and voter turnout across European countries and found a strong gradient, with the association only becoming marked among participants over the age of 50. Corroborating this finding, studies focused on older age groups tend to find higher magnitudes of association for disability and self-rated health measures (Bazargan, Kang, & Bazargan, 1991; Schur et al., 2002, Burden et al. 2016).

There is strong reason to believe that changes in health influence voting through different mechanisms at different ages. Examining the voting practices of young adults across US elections, Plutzer (2002) demonstrated that the determinants of initial voting experiences were fundamentally distinct from those of subsequent voting experiences over the course of adulthood. Building on this theoretical argument, Ojeda (2015, Ojeda & Pacheco, 2019) proposed two sets of mechanisms to understand the role of health on voting over the life-course. Before the entry into adulthood, health is hypothesized to influence the capacity to acquire in one's family and school the resources enabling political participation. These include general cognitive skills, political knowledge and interest, feelings of affiliation to a political party, and the establishment of social networks that may reinforce these resources over time. Once resources and initial voting experiences are acquired, health is then hypothesized to influence voting by disrupting a new set of resources driving the capacity to vote, such as knowledge of one's environment, social networks, and physical functioning.

Other life-course processes may shape the relationship between health and voting over time. Period effects, such as the importance or closeness of one election, strongly drive voter turnout (Cancela & Geys, 2016; Frenk, Yang, & Land, 2013). Countries, including Canada, Norway, Denmark, the US, and the UK, have also been facing widening inequalities in voter turnout across generations (Kitanova, 2019; Smets, 2012). Smets and Neundorf (2014) argued that these period and cohort effects could also interact, finding in the US that cohorts were more likely to vote in adulthood if they experienced their initial voting experiences in elections with high turnout rates. It is therefore possible that differences in voting attributable to health at different ages are exacerbated in electoral contexts where the resources enabling the capacity to vote are more likely to matter, that is, when elections are less important and among younger generations who are less likely to vote.

### 1.3 Objectives

Except for the work among US young adults by Ojeda and Pacheco (2019), no study that we know of has disentangled the relationship between health and voting over the course of adulthood in a longitudinal approach. Building on the developmental approach to lifelong voting, we seek to test the hypotheses that: 1) health is associated with initial voting experiences, 2) beyond initial voting experiences, health continues to be associated with voting in adulthood; 3) health has an increased association with voting as individuals become older. In keeping with a life-course approach, we hypothesize that declines in health have an increasing impact on voter turnout as they cumulatively impact on the resources driving the capacity to vote over the course of adulthood. To examine this, the current study examines the progression of the health-voting association using two British birth cohorts, the 1958 National Child Development Study (NCDS) and the 1970 British Cohort study (BCS), which have already been used to study the determinants of voter turnout (Denny & Doyle, 2005, 2007a, 2008; Deary, Batty, & Gale, 2008; Finlay & Flanagan, 2013; Persson, 2014).

## Methods

### 2.1 Data

We used data from the 1958 NCDS, which recruited 17,415 individuals from birth and followed them up to the ages of 55 in 2013, and the 1970 BCS, which recruited 17,196 individuals from birth and followed them up to the age of 42 in 2012 (Power & Elliott, 2006; Elliott & Shepherd, 2006; Chamberlain et al. 2013, University of London, 2008, University of London, 2008, University of London, 2008, University of London, 2008, University of London, 2012, University of London, 2014, University of London, 2015, University of London, 2016a, University of London, 2016b, University of London, 2016c, University of London, 2016d, University of London, 2016e, University of London, 2019). The 1958 and 1970 cohorts were initially designed to study perinatal mortality and then progressed to become multidisciplinary, collecting information on health, economic, and social circumstances over time. We used the data on participants who reported on voting in the last general election at least once in all valid waves: in the 1958 cohort, six times at the ages of 23, 32, 42, 46, 50, and 55 (14,031 participants); in the 1970 cohort, four times at the ages of 30, 34, 38, and 42 (12,973 participants). While the 1970 cohort was followed for a relatively shorter period of time, its participants voted in the same elections from 1997 to 2010 as the 1958 cohort, enabling us to consider here potential period and cohort effects.

### 2.2 Measures

For the dependent variable, participants were asked whether they voted in the last general election (Yes/No), referring: 1) in the 1958 NCDS, to the 1979 (age 23), 1987 (age 32), 1997 (age 42), 2001 (age 46), 2005 (age 50), and 2010 (age 55) general elections; 2) in the 1970 BCS, to the 1997 (age 30), 2001 (age 34), 2005 (age 38), and 2010 (age 42) general elections. The 1979 election represented the first election in which the 1958 cohort could vote while the 1997 election represented the second election in which the 1970 cohort could vote. Missingness on voter turnout was relatively marked in the 1970 cohort at the ages of 38 (21.5%) and 42 (13.3%) because these measures were taken retrospectively at the age of 42 from a questionnaire completed by 89% of participants alongside their main interview.

To operationalize health, we chose two indicators consistently measured across survey waves in the 1958 and 1970 cohorts: self-rated health and longstanding limitations. For self-rated health, participants were asked to rate their health using different four- or five-point Likert-type scales across waves, with options ranging from “very poor” to “excellent”. We recoded responses into three consistent categories: 1) Good to excellent, 2) Fair, and 3) Poor to very poor. For longstanding limitations, participants were asked through various questions whether they felt limited in their daily activities when they reported having a longstanding illness and/or disability (Yes/No). We chose these indicators to represent different facets of health: in particular, self-rated health may indicate shorter-term problems and limitations may indicate longer-term conditions that require social and material adaptations (Stockemer & Rapp, 2019). Question labels and responses on voting and health measures are presented in Supplementary Table 1.

To address confounding, we selected a group of variables that were available in each cohort, consistently measured across waves, and likely to be associated with health and voting (Smets & van Ham, 2013). Characteristics at birth included: 1) gender (using sex as a proxy) (Man/Woman); 2) region; 3) mother's age (continuous); 4) mother's smoking during the pregnancy (Yes/No); 5) mother's weight (continuous); 6) father's Registrar General's social class (I Professional/II Managerial and technical/III Skilled/IV or V Partly-skilled or unskilled/Not applicable); and 7) birth weight (continuous). Characteristics at the ages of 23/30 included: 8) intention to vote in the next election (Yes/No/Do not know); 9) educational attainment (No qualifications/NVQ 1: CSE level/NVQ 2: O level/NVQ 3: A level/NVQ 4: Higher qualification/NVQ 5: Degree). Time-varying characteristics in adulthood included: 10) Registrar General's social class (I/II/III/IV or V/Not applicable), 11) employment status (Employed/Unemployed/Homemaker/Other); 12) parenthood (No children/One child/Two or more children); 13) marital status (Single/Married or partnered/Separated, divorced, or widowed); 14) housing tenure (Main owner/Renter/Other). The Registrar General's scheme allocated people to social classes based on employment and occupation, was found to be generally strongly correlated with income, and has been the most common measure of social class in the UK until the early 2000s (Goldthorpe, 2010, Office for National Statistics, 2019).

### 2.3 Statistical analyses

Before our main analyses, we modelled inverse-probability non-response weights for each wave using participants' circumstances at birth (Hawkes & Plewis, 2006; Mostafa & Wiggins, 2015). British birth cohort members are not rejected when they do not answer in one wave. They are re-contacted at following waves unless they refuse to participate. This means that missingness patterns are non-monotonous. Predictors of non-response included: being a man, having no record of the father's social class, having a lower birth weight, and having a mother who was younger, with a lower weight, and who smoked during the pregnancy.

For our main analyses, we examined the adjusted associations between the two health indicators and voting using random-effects (RE) logistic models. One of the strengths of RE models is their ability to derive unbiased estimates of person-specific effects in the presence of non-response during the follow-up period, under the condition that it is missing-completely-at-random (MCAR) (Gibbons, Hedeker, & DuToit, 2010). Since only 40% of the 1958 cohort (*n* = 5578 out of 14,301) and 52% of the 1970 cohort (*n* = 6700 out of 12,973) participants answered to each wave during the follow-up period, this approach maximizes the data available for analysis.

We did not use fixed-effects (FE) modelling despite its capacity to account for time-invariant confounding because of the high variability required in the outcome. Since only 43% of the 1958 cohort and 30% of the 1970 cohort participants with valid data on voting reported both outcomes (i.e., voting and not voting) over the period of follow-up, we argue that this approach would limit the capacity to detect significant differences, especially in the relatively smaller group of participants who reported poor health. We also note that we were unable to implement a multiple imputation (MI) approach to reduce the impact of data-missing-at-random (MAR) because, given the longitudinal nature of our data and the large number and nominal scale of our variables, we faced well-known irreconcilable convergence issues when testing models (De Silva, Moreno-Betancur, De Livera, Lee, & Simpson, 2017). Our analysis therefore builds on the assumption that there is no systematic difference between observations with complete and incomplete data within waves (De Silva et al., 2017).

We first estimated the average association of the health indicators with voting across waves in each cohort. We modelled health indicators and covariates in sequential blocks to assess their contribution: Model 1 – Bivariate, Model 2 – Health variables together, Model 3 – Adding characteristics at birth, Model 4 – Adding characteristics at ages 23/30, Model 5 (Full) – Adding time-varying characteristics. Detailed results from the full models are presented in Supplementary Table 2. We then estimated the potential age-based differences in these associations by entering additional age-based interaction terms with the two health indicators after the full model. During the second step, we also produced marginal probabilities to better interpret differences in voting across waves (Muller & MacLehose, 2014).

We tested interactions between the two health indicators and between each indicator and sex, and found no additional statistically significant interactions. We also tested if results varied when considering: 1) the number of waves in which cohort members participated (reproducing analyses in sub-samples of cohort members who participated in at least one, two, three, or more waves in each cohort), 2) non-response weights, and 3) intention to vote at ages 23/30, and obtained consistent results (see Supplementary Table 3). Analyses were produced in each dataset separately using Stata 14 (StataCorp 2015).

## 3 Results

### 3.1 Sample characteristics

Table 1 presents the frequencies related to self-rated health, limitations in everyday activities, and voter turnout in the 1958 and 1970 cohorts. Weighting for non-response, voting in the last general election varied: 1) in the 1958 cohort, between 67% and 77% at the ages of 23, 32, 42, 46, 50, and 55; 2) in the 1970 cohort, between 62% and 73% at the ages of 30, 34, 38, and 42. The decrease in voting after the age of 42 in the 1958 cohort and the lower prevalence of voting in the 1970 cohort are likely to be explained by the historical drop in voter turnout around the 2001 UK general election (Dempsey & Loft, 2017). The estimated prevalence of fair or poor self-rated health varied: 1) in the 1958 cohort, between 10% and 24% between the ages of 23 and 55; 2) in the 1970 cohort, from 15% to 21% between the ages of 30 and 42. The estimated prevalence of limitations in everyday activities varied: 1) in the 1958 cohort, from 2% to 20% between the ages of 23 and 55; 2) in the 1970 cohort, between 9% and 17% between the ages of 30 and 42. Comparing these indicators across cohorts at the age of 42, the 1970 cohort was slightly more likely to be in good or better health (85% versus 82%) yet slightly more likely to report limitations in everyday activities (17% versus 13%).

**Table 1:** Sample characteristics of the 1958 and 1970 cohort studies.

		General election year											
		1979		1987		1997		2001		2005		2010	
		N	%	N	%	N	%	N	%	N	%	N	%
**1958 NCDS**	**Age**	23		32		42		46		50		55	
	**Sample size**	11,889		10,899		10,830		9057		9279		8670	
	**Voter turnout**												
	Did not vote	3.931	33.1	2465	22.6	2468	22.8	2119	23.4	2456	26.5	2193	25.3
	Voted	7890	66.4	8314	76.3	8298	76.6	6866	75.8	6678	72.0	6161	71.1
	*Missing*	68	0.6	120	1.1	64	0.6	72	0.8	145	1.6	316	3.6
	**Self-rated health**												
	Good to excellent	10,742	90.4	9258	84.9	8838	81.6	6944	76.7	7518	81.0	6864	79.2
	Fair	1029	8.7	1268	11.6	1567	14.5	1438	15.9	1177	12.7	1196	13.8
	Poor or very poor	106	0.9	191	1.8	382	3.5	668	7.4	530	5.7	517	6.0
	*Missing*	12	0.1	182	1.7	43	0.4	7	0.1	54	0.6	93	1.1
	**Limitations in everyday activities**												
	No	11,324	95.3	10,118	92.8	9346	86.3	8095	89.4	7804	84.1	6974	80.4
	Limited	246	2.1	676	6.2	1438	13.3	954	10.5	1462	15.8	1684	19.4
	*Missing*	319	2.7	105	1.0	46	0.4	8	0.1	13	0.1	12	0.1

**1970 BCS**	**Age**	–		–		30		34		38		42	
	**Sample size**					10,442		8961		8232		9116	
	**Voter turnout**												
	Did not vote					3906	37.4	3274	36.5	1510	18.3	2047	22.5
	Voted					6464	61.9	5614	62.7	4953	60.2	5856	64.2
	*Missing*					72	0.7	73	0.8	1769	21.5	1213	13.3
	**Self-rated health**												
	Good to excellent					8830	84.6	7049	78.7	7283	88.5	7695	84.4
	Fair					1334	12.8	1321	14.7	678	8.2	969	10.6
	Poor or very poor					231	2.2	564	6.3	241	2.9	415	4.6
	*Missing*					47	0.5	27	0.3	30	0.4	37	0.4
	**Limitations in everyday activities**												
	No problem					9469	90.7	8303	92.7	7524	91.4	7608	83.5
	Limited					922	8.8	658	7.3	705	8.6	1502	16.5
	*Missing*					51	0.5	0	0	3	0.0	6	0.1

### 3.2 Health and voting over the course of adulthood: main associations

The first step was to examine the average associations between health and voting across waves in the 1958 and 1970 cohorts. Supporting our first hypothesis, we found that rating one's health as fair or poor was associated with a lower probability of voting in adulthood. Having limitations in everyday activities, however, was not associated with voting after taking into account self-rated health. The magnitude of associations was overall slightly stronger in the 1970 cohort. Table 2 presents the results of the random-effects models estimating these associations in the two cohorts.

**Table 2:** Health and voting in the 1958 and 1970 cohorts: main effects.

		Model 1 Separately		Model 2 Together		Model 3 + Birth		Model 4 + Ages 23/30		Model 5 + Time-varying	
		OR	95%CI	OR	95%CI	OR	95%CI	OR	95%CI	OR	95%CI
**NCDS 1958**	**Self-rated health**										
	Fair	**0.73**	**(0.68**–**0.80)**	**0.74**	**(0.68**–**0.81)**	**0.76**	**(0.70**–**0.83)**	**0.82**	**(0.75**–**0.91)**	**0.85**	**(0.77**–**0.94)**
	Poor or worse	**0.61**	**(0.52**–**0.70)**	**0.62**	**(0.53**–**0.72)**	**0.67**	**(0.57**–**0.78)**	**0.75**	**(0.63**–**0.89)**	0.83	(0.69–1.00)
	(Good or better = ref.)										
	**Limitations in everyday activities**										
	Limited (No = ref.)	**0.81**	**(0.74**–**0.90)**	0.97	(0.88–1.08)	0.96	(0.87–1.07)	1.02	(0.91–1.15)	1.11	(0.98–1.25)

**BCS 1970**	**Self-rated health**										
	Fair	**0.67**	**(0.60**–**0.76)**	**0.68**	**(0.60**–**0.77)**	**0.68**	**(0.59**–**0.78)**	**0.77**	**(0.67**–**0.88)**	**0.82**	**(0.72**–**0.95)**
	Poor or worse										
	(Good or better = ref.)	**0.51**	**(0.41**–**0.63)**	**0.52**	**(0.41**–**0.66)**	**0.54**	**(0.42**–**0.69)**	**0.65**	**(0.50**–**0.84)**	**0.68**	**(0.52**–**0.90)**
	**Limitations in everyday activities**										
	Limited (No = ref.)	**0.79**	**(0.67**–**0.92)**	0.95	(0.80–1.13)	0.95	(0.78–1.15)	1.02	(0.84–1.24)	1.07	(0.87–1.31)

Estimates represent odds ratios (OR) from weighted random-effects logistic models. Bolded estimates are statistically significant at the *p* < .05 level. Models 1–5 controlled for age. Model 3 included: gender, region, mother's age, mother's smoking, mother's weight, father's social class, birth weight. Model 4 included: intention to vote in the next election, educational attainment. Model 5 included: social class, employment status, parenthood, marital status, housing tenure.

In the 1958 cohort, participants who rated their health as fair had 15% lower odds of voting (95%CI 0.77-0.94) and participants who rated their health as poor had 17% lower odds of voting (95%CI 0.69-1.00) compared to those who rated their health as good to excellent. Comparing estimates from Models 1 and 5 on the log odds scale, the magnitude of the estimates related to self-rated health decreased by 48% (fair) and 62% (poor). Reporting limitations in everyday activities was not significantly associated with voting in the final model (OR = 1.11, 95%CI 0.98-1.25). Other covariates associated with voting in the 1958 cohort included: being a woman, being older, having a father in a higher social class, having an older mother, having a higher level of education, intending to vote in the next election, being employed, being married, having no children, and being a home owner (see online supplementary material).

In the 1970 cohort, participants who rated their health as fair had 18% lower odds of voting (95%CI 0.72-0.95) and participants who rated their health as poor had 32% lower odds of voting (95%CI 0.52-0.90) compared to those who rated their health as good to excellent. Comparing estimates from Models 1 and 5 on the log odds scale, the magnitude of the estimates related to self-rated health decreased by 51% (fair) and 42% (poor). Reporting limitations in everyday activities was not significantly associated with voting in the final model (OR = 1.07, 95%CI 0.87-1.31). Other covariates associated with voting in the 1970 cohort included: being older, having an older mother, having a higher birth weight, having a higher level of education, intending to vote in the next election, having a higher social class, being married, and being a home owner (see online supplementary material).

### 3.3 Health and voting over the course of adulthood: age-graded associations

The next step was to investigate potential differences in the associations between the two health indicators and voting at different ages over the course of adulthood. Testing age-based interactions terms with the full models (Model 5) in the 1958 and 1970 cohorts, we found evidence of change in the two cohorts. In the 1958 cohort, using the latest time point (age 55) as the reference category: 1) the interaction term for “poor or worse” self-rated health was significant at the age of 23 (*p* = .025); 2) the interaction terms for limitations in everyday activities were significant at the ages of 23 (*p* = .005) and 32 (*p* = .015). In the 1970 cohort, using the latest time point (age 42) as the reference category: 1) the interaction terms for “fair” (*p* = .014) and “poor or worse” (*p* = .027) self-rated health were significant at the age of 34; 2) the interaction term for limitations in everyday activities (*p =* .013) was significant at the age of 30. Contrasting with our first and second hypotheses, we found that: 1) there was no clear age-graded increase in the relationship between self-rated health and voting in the life period covered in the 1958 and 1970 cohorts; 2) there was a positive association between limitations in everyday activities and voting at the age of 55 in the 1958 cohort and at the age of 30 in the 1970 cohort.

Table 3 presents the age-graded marginal probabilities of voting according to health indicator categories across waves in each cohort. In the 1958 cohort, the difference in the probability of voting between those who reported “fair” and “good or better” health varied from +0.1 p.p. to - 3.5 p.p. between the ages of 23–55. The difference in the probability of voting for those who reported “poor or worse” health was most marked at the age of 23 (13.4 p.p.), and returned afterwards to similar levels of differences as found in those in fair health, varying from - 0.1 p.p. to - 3.0 p.p., between the ages of 32 and 55. Differences in voting according to limitations in everyday activities changed direction with age, starting from a disadvantage of - 5.7 p.p. at the age of 23 to an advantage of +3.3 p.p. at the age of 55.

**Table 3:** Health and voting in the 1958 and 1970 cohorts: age-based effects.

		General election year					
		1979	1987	1997	2001	2005	2010
		%	%	%	%	%	%
**1958 NCDS**	**Age**	23	32	42	46	50	55
	**Self-rated health**						
	Good to excellent	**68.8**	77.9	**77.8**	**76.1**	73.1	73.0
	Fair	67.3	78.0	**74.2**	**73.2**	70.9	70.9
	Poor or very poor	**55.3**	77.2	75.4	75.1	70.1	71.8
	**Limitations in everyday activities**						
	Limited	62.6	75.9	78.7	78.2	73.8	**75.5**
	No problem	68.3	77.9	77.0	75.5	72.6	**72.3**

**1970 BCS**	**Age**	–	–	30	34	38	42
	**Self-rated health**						
	Good to excellent			**65.1**	63.7	**74.6**	**74.3**
	Fair			62.6	63.7	**70.8**	**69.2**
	Poor or very poor			**56.5**	62.0	69.8	**65.7**
	**Limitations in everyday activities**						
	Limited			**68.7**	66.1	71.6	73.7
	No problem			**64.2**	63.6	74.3	72.5

Estimates are marginal probabilities from the full models (Model 5) presented in Table 2, with age-based interactions. Interaction terms for self-rated health and limitations in everyday activities were entered separately. Differences between bolded estimates are statistically significant at the *p* < .05 level.

In the 1970 cohort, the difference in the probability of voting between those who reported “fair” and “good or better” health varied from 0.0 p.p. to - 5.1 p.p. between the ages of 30–42. The differences in the probability of voting for those who reported “poor or worse” health were marked both at the ages of 30 (- 8.5 p.p.) and 42 (- 8.7 p.p.). Differences in voting according to limitations in everyday activities also followed an age-based pattern, starting from a benefit of +4.5 p.p. at the age of 30 to a disadvantage of - 1.2 p.p. at the age of 42.

## 4 Discussion

Our study sought to corroborate the role of health on voting over the course of adulthood, making use of two British birth cohort studies spanning three decades of life. Supporting our first hypothesis and the bulk of the studies that have examined this question, above the influence of family background and adult achievements, we found a significant negative association with self-rated health, but not with health limitations in everyday activities, and voting in the 1958 and 1970 cohorts. Comparing the magnitude of its effect with other covariates, we find that the importance of self-rated health was comparable to the predictive power of social class, parenthood, marriage, and employment, supporting the argument that health may be more important for understanding political behaviour than previously believed (Mattila et al., 2013, Gollust & Rahn, 2015).

Returning to our first objective, we found that the health-voting relationship was robust to a number of circumstances at birth, young adulthood, and midlife. While some early-life circumstances (father's social class, mother's age, birth weight) were significantly associated with voting, they did not appear to influence the magnitude of the health-voting relationship. Between 42% and 62% of this association, however, was attenuated when considering intention to vote, educational attainment, employment, marital status, parenthood, and home ownership over the course of adulthood. Additionally, our analytic approach did not explicitly disentangle the nature of the relationships between self-rated health, other time-varying characteristics, and voting. While poor health is considered to be the result of unfavourable circumstances accumulating over the life-course, it also reinforces disadvantage over the life-course. Poor self-rated health may therefore influence voting by limiting the opportunities to secure relationships and home ownership, which are positively linked with voter turnout (Franke & Kulu, 2017; Smith, 2012). This selection effect would lead us to under-estimate the full size of the health-voting association.

Regarding our second objective, improving on other studies with the use of an age-graded analysis in two cohorts that experienced the same elections, we were able to highlight a considerable variation in the magnitude of the association between self-rated health and voting across waves and cohorts. We found that this association was strongest in the first election captured at the age 23 wave in the 1958 cohort and overall slightly stronger in the 1970 cohort. This supports the argument that there are likely to be multiple life-course moderators nuancing this relationship.

Following the work of Plutzer (2002) and Ojeda and Pacheco (2019), we found in the 1958 cohort that poor health was most likely to influence voting among participants during their first experience voting in a general election in young adulthood. Similar results found in the second experience voting in a general election (captured at the age 30 wave) in the 1970 cohort support the important role that poor health may play during this life period. In comparison, the association between health and voting was absent during the third opportunity to vote in a general election in both cohorts (captured at age 32 in the 1958 cohort and at age 34 in the 1970 cohort). This suggests that health may be important in shaping voting habits during young adulthood yet decreasingly so in subsequent elections, supporting the findings by Ojeda and Pacheco (2019) in the US. Supporting this in the context of general elections, Smets and Neundorf (2014) found that the first two elections young people could participate in were the most critical in shaping their long-term voting trajectories. Since the voting habit is one of the strongest predictors of voting in future elections, a lower propensity to vote attributable to health may disproportionally lead unhealthy young adults into long-term trajectories of disengagement (Denny & Doyle, 2009). New studies will need to confirm whether these findings are consistent across other instances of voting (local, regional, European) that young adults encounter alongside national elections.

Beyond age, we found little evidence supporting substantial period effects when comparing results across elections. For instance, while health was a relatively distant second priority in the 2010 UK election in keeping with the high level of satisfaction with the UK health care system, our estimates suggest that those in poor health were only 2% less likely to vote if they were in the 1958 cohort yet 12% less likely to vote if they were in the 1970 cohort (King’s Fund 2010). We found, however, more evidence leading to the consideration of a cohort effect as participants reported a relatively slightly lower propensity to vote if they were in poor health in the 1970 cohort. This finding contributes to the argument that voter turnout in the younger generations that demonstrate political disengagement and dissatisfaction in politics (as was the 1970 cohort around the 2001 UK election) may be shaped to a higher degree by the individual resources driving the capacity to vote, including health (Dempsey & Loft, 2017).

Finally, we return to the relatively surprising finding that, controlling for self-rated health and other covariates, reporting limitations in everyday activities was positively associated with voting in the third (1970 cohort) and fifth decades (1958 cohort) of life. It is possible that, compared to self-rated health, the measure of health limitations taps to a lesser extent into the dimensions of health that has a strong influence on the capacity to vote such as physical vitality (Cohen, Forbes, & Garraway, 1995; Mavaddat et al., 2011). Stockemer and Rapp (2019) used a similar measure of health limitations in the European Social Survey and found that it was associated with a lower probability of voting but a higher probability of contacting a politician, signing a petition, wearing a campaign badge, and boycotting in the past year. Two studies in the US and Finland also found that, while many chronic conditions were associated with a decreased voter turnout, individuals with cancer had a higher probability of voting (Gollust & Rahn, 2015; Sund et al., 2017). Finding that finances, physical vitality, and social networks were unlikely to mediate this association, Gollust and Rahn (2015) hypothesized that higher voter turnout could be related to the development of an activist identity related to health conditions, leading those who strongly associate with it to further engage in political processes.

One key issue in interpreting this finding resides in our limited ability to unpack the nature of health limitations. In particular, this measure may disproportionally include individuals with milder forms of disability. Studies on disability have consistently found that: 1) associations between disability and voting remain negative over time, 2) they are robust to the nature of the limitation measured (e.g., hearing, visual, cognitive, mobility), and 3) they are age-graded and most marked among older adults (Schur et al. 2002, 2005, Matsubayashi & Ueda, 2014). Future studies will need to unpack which limiting conditions are likely to impact voting and whether these fit with an identity hypothesis (Gollust & Rahn, 2015).

### 4.1 Strengths and limitations

This study builds on the qualities of the British birth cohorts to produce representative estimates of the role of health on voting in adulthood across two generations in the United Kingdom. This includes the capacity to use a large set of variables at different life-course stages to account for participants’ family background and changing social circumstances. We highlight a few limitations. First, concerns about unobserved heterogeneity remain plausible, precluding us from interpreting causal relations. We tested modelling additional measures of limitations at the age of 16 and political interest in adult waves, and found that they did not influence our findings. Second, we did not use an analytic strategy to adjust for item-level missingness due to issues of non-convergence (De Silva et al., 2017). Non-random missingness may therefore bias our findings. Third, differences in the measurement of health indicators over the course of the cohorts led us to use fewer categories, which have underestimated differences between extreme categories. Finally, the 1958 and 1970 cohorts were not designed to study voter turnout and waves have been administered years after the last general election. Our findings build on the assumption that variables were valid proxies of the circumstances experienced when the elections occurred.

### 4.2 Conclusion

Voting is a central element of social cohesion and democracy that is shaped by health. This relationship is not straightforward and may include both the lower propensity to vote with poorer health and the higher propensity to vote when health limits everyday activities. These relationships emerge at different ages, with some of the strongest associations likely to be found during habit formation in young adulthood. Contrasting with the current generation, young adults in poor health today may be even less likely to vote compared to the 1958 and 1970 cohorts as they further delay their transition to adulthood and their participation in politics (Smets, 2016). The findings support the argument that health impacts voting behaviour, and thus elections, long before it may lead to death (Rodriguez, 2018; Rodriguez et al., 2015). The findings also support the argument that those in poor health are likely to suffer a lack of political power over their life-course, which in turn contribute to the reproduction of social inequalities in voting and health (Gollust & Rahn, 2015; Pacheco & Fletcher, 2014). Next research steps include disentangling this association beyond the age of 55 in a longitudinal setting, extending its study to other dimensions of health such as mental wellbeing, and formally testing its contribution in socioeconomic inequalities in voting.

## Ethics statement

There was no need to obtain ethics approval for the secondary data analysis of publicly available data in this study. For the 1958 cohort, ethical approval for 1958–1991 and 2004 was gained by internal review only; for 2000 and 2008 by the London multicenter research ethics committee (MREC); for 2002 by the South-East MREC; and for 2013 by the London-Central MREC. For the 1970 cohort, ethical approval for 1970–1996 and 2004 was gained by internal review only; for 2000 by the London MREC; for 2008 by the Southampton and South West Hampshire MREC; for 2012 by the London-Central MREC.

## CRediT authorship contribution statement

**Thierry Gagné:** Conceptualization, Methodology, Formal analysis, Writing - original draft, Writing - review & editing. **Ingrid Schoon:** Methodology, Writing - review & editing, Supervision. **Amanda Sacker:** Methodology, Writing - review & editing, Supervision.
