# Supplementary material for: Health and voting over the course of adulthood: Evidence from two British birth cohorts
Source: SSM Popul Health. 2019 Dec 16;10:100531. doi: 10.1016/j.ssmph.2019.100531 (PMC7211898; doi:10.1016/j.ssmph.2019.100531)
Supplement: Multimedia component 1 [file mmc1.docx]

**SUPPLEMENTARY TABLE 1**

Variable labels and responses

| **Variable** | **1958/1970** | **Age** | **Question** | **Responses** |
| --- | --- | --- | --- | --- |
|  |  |  |  |  |
| **Self-rated health** | 1958 | 23 | How would you describe your health generally? Would you say it is… | 1) Excellent  2) Good  3) Fair  4) Poor |
|  | 1958 | 32 | How would you describe your health generally? Would you say it is… | 1) Excellent  2) Good  3) Fair  4) Poor |
|  | 1958 | 42 | How would you describe your health generally? Would you say it is… | 1) Excellent  2) Good  3) Fair  4) Poor |
|  | 1958 | 46 | Please think back over the last 12 months about how your health has been. Compared to people of your own age, would you say that your health has on the whole been... | 1) Excellent  2) Good  3) Fair  4) Poor  5) Very poor |
|  | 1958 | 50 | In general, would you say your health is… | 1) Excellent  2) Very good  3) Good  4) Fair  5) Poor |
|  | 1958 | 55 | In general, would you say your health is… | 1) Excellent  2) Very good  3) Good  4) Fair  5) Poor |
|  | 1970 | 30 | How would you describe your health generally? Would you say it is… | 1) Excellent  2) Good  3) Fair  4) Poor |
|  | 1970 | 34 | Please think back over the last 12 months about how your health has been. Compared to people of your own age, would you say that your health has on the whole been... | 1) Excellent  2) Good  3) Fair  4) Poor  5) Very poor |
|  | 1970 | 38 | In general, would you say your health is… | 1) Excellent  2) Very good  3) Good  4) Fair  5) Poor |
|  | 1970 | 42 | In general, would you say your health is… | 1) Excellent  2) Very good  3) Good  4) Fair  5) Poor |
|  |  |  |  |  |
| **Health affects everyday activities** | 1958 | 23 | Five questions: How much does your condition limit your activities? From this card, please tell me how much difficulty, if any, you have in… 1) washing or dressing yourself?; 2) getting about the house?; 3) doing housework?; 4) getting out of the house on your own?; 5) leading your social life? | 1) Yes  2) No |
|  | 1958 | 32 | (For up to four different long-standing illness, disability, or infirmity conditions) Does this health problem limit your daily activities in any way compared to people of your own age? | 1) Yes  2) No |
|  | 1958 | 42 | (For up of ten potential different long-standing illness, disability, or infirmity condition) Does this condition limit your daily activities in any way compared to people of your own age? | 1) Yes  2) No |
|  | 1958 | 46 | Does your health in any way limit your daily activities compared to most people of your age? | 1) Yes  2) No |
|  | 1958 | 50 | Does your health in any way limit your daily activities compared to most people of your age? | 1) Yes  2) No |
|  | 1958 | 55 | Do any of your conditions or illnesses reduce your ability to carry out day-to-day activities? | 1) Yes  2) No |
|  | 1970 | 30 | (For up of ten potential different long-standing illness, disability, or infirmity condition) Does this condition limit your daily activities in any way compared to people of your own age? | 1) Yes  2) No |
|  | 1970 | 34 | Does your health in any way limit your daily activities compared to most people of your age? | 1) Yes  2) No |
|  | 1970 | 38 | Does your health in any way limit your daily activities compared to most people of your age? | 1) Yes  2) No |
|  | 1970 | 42 | Do any of your conditions or illnesses reduce your ability to carry out day-to-day activities? | 1) Yes  2) No |
| **Voter turnout** | 1958 | 23 | Did you vote in the last General Election in May 1979? | 1) Yes  2) No |
|  | 1958 | 32 | Did you vote in the last General election in June 1987? | 1) Yes  2) No |
|  | 1958 | 42 | Did you vote in the last General election, in May 1997? | 1) Yes  2) No |
|  | 1958 | 46 | Did you vote in the last General election in June 2001? | 1) Yes  2) No |
|  | 1958 | 50 | Did you vote in the last General election in May 2005? | 1) Yes  2) No |
|  | 1958 | 55 | Did you vote in the last General election in May 2010? | 1) Yes  2) No |
|  | 1970 | 30 | Did you vote in the last General election, in May 1997? | 1) Yes  2) No |
|  | 1970 | 34 | Did you vote in the last General election in June 2001? | 1) Yes  2) No |
|  | 1970 | 38 | Which party did you vote for in the General Election in May 2005? (Responses were the following: Conservative; Labour; Liberal Democrats; Plaid Cymru; Scottish National Party; Green Party; UK Independence Party; Other; Did not vote) | 1) Yes  2) No |
|  | 1970 | 42 | Which party did you vote for in the General Election in May 2010? (Responses were the following: Conservative; Labour; Liberal Democrats; Plaid Cymru; Scottish National Party; Green Party; UK Independence Party; Other; Did not vote) | 1) Yes  2) No |

**SUPPLEMENTARY TABLE 2**

Health and voting in the 1958 and 1970 cohorts: main effects

|  |  | Full model  1958 NCDS | | Full model  1970 BCS | |
| --- | --- | --- | --- | --- | --- |
| Construct | Variable | OR | 95%CI | OR | 95%CI |
|  |  |  |  |  |  |
| **Health indicators** | **Self-rated health**  Fair  Poor or worse  (Good or better = ref.) | **0.85**  0.83 | **(0.77-0.94)**  (0.69-1.00) | **0.82**  **0.68** | **(0.72-0.95)**  **(0.52-0.90)** |
|  | **Limitations in everyday activities** |  |  |  |  |
|  | Limited  (No = ref.) | 1.11 | (0.98-1.25) | 1.07 | (0.87-1.31) |
|  |  |  |  |  |  |
| **Sweep** | **Sweep** (see below) |  |  |  |  |
| **Birth** | **Sex**  Woman  (Man = ref.) | **1.16** | **(1.06-1.27)** | 1.12 | (0.99-1.26) |
|  | **Region** (see below) |  |  |  |  |
|  | **Smoking during pregnancy**  Yes  (No = ref.) | 0.93 | (0.85-1.02) | 1.05 | (0.93-1.19) |
|  |  |  |  |  |  |
|  | **Father’s social class**  II  III  IV or V  Not applicable  (I = ref.) | 0.93  0.80  **0.73**  **0.71** | (0.72-1.19)  (0.64-1.00)  **(0.57-0.93)**  **(0.53-0.96)** | 0.91  0.79  0.77  0.84 | (0.67-1.23)  (0.60-1.03)  (0.57-1.04)  (0.59-1.19) |
|  |  |  |  |  |  |
|  | **Mother’s weight**  Continuous (1958: stones / 1970: kilograms) | 0.98 | (0.95-1.01) | 1.01 | (0.99-1.01) |
|  | **Mother’s age**  Continuous (years) | **1.01** | **(1.00-1.02)** | **1.02** | **(1.01-1.03)** |
|  | **Birth weight**  Continuous (1958: ounces / 1970: grams) | 0.99 | (0.99-1.00) | **0.99** | **(0.99-0.99)** |
| **Age 23/30** | **Educational attainment** |  |  |  |  |
|  | NVQ 1  NVQ 2  NVQ 3  NVQ 4  NVQ 5  (No academic qualification = ref.) | **1.60**  **2.04**  **3.02**  **2.71**  **3.89** | **(1.36-1.87)**  **(1.79-2.32)**  **(2.57-3.53)**  **(2.26-3.25)**  **(3.18-4.75)** | 1.16  **1.50**  **1.92**  **2.67**  **2.69** | (0.90-1.49)  **(1.24-1.81)**  **(1.53-2.41)**  **(2.16-3.29)**  **(1.93-3.74)** |
|  | **Intent to vote in next election**  Will vote  Will not vote  (Does not know = ref.) | **1.51**  **0.24** | **(1.29-1.78)**  **(0.20-0.29)** | **1.54**  **0.09** | (1.25-1.90)  (0.07-0.12) |
| **Time-varying** | **Social class** |  |  |  |  |
|  | II  III  IV or V  Not applicable  (I = ref.) | 0.90  0.85  0.84  **0.80** | (0.74-1.08)  (0.70-1.02)  (0.69-1.03)  **(0.62-0.98)** | 0.89  **0.75**  **0.75**  **0.62** | (0.71-1.10)  **(0.60-0.94)**  **(0.58-0.98)**  **(0.40-0.97)** |
|  | **Employment status** |  |  |  |  |
|  | Unemployed  Homemaking  Other  (Employed = ref.) | 0.94  1.05  0.89 | (0.80-1.11)  (0.92-1.21)  (0.74-1.06) | 1.47  1.17  **1.44** | (0.91-2.38)  (0.77-1.80)  **(1.00-2.06)** |
|  |  |  |  |  |  |
|  | **Marital status**  Married  Widowed/Divorced/Separated  (Single = ref.) | **1.21**  **0.68** | **(1.08-1.36)**  **(0.59-0.78)** | **1.58**  0.93 | **(1.40-1.79)**  (0.78-1.12) |
|  |  |  |  |  |  |
|  | **Parenthood**  One child  Two or more children  (No children = ref.) | **0.86**  0.91 | **(0.77-0.97)**  (0.81-1.01) | 1.07  1.03 | (0.93-1.23)  (0.89-1.18) |
|  |  |  |  |  |  |
|  | **Housing tenure**  Owner  Other  (Renter = ref.) | **1.51**  **1.40** | **(1.36-1.66)**  **(1.23-1.59)** | **1.62**  1.22 | **(1.43-1.84)**  (0.99-1.48) |

Bolded estimates are statistically significant at the *p* < .05 level*.*

**Results for sweep and region**

|  | Full model  1958 NCDS | |  | Full model  1970 BCS | |
| --- | --- | --- | --- | --- | --- |
| Variable | OR | 95%CI | Variable | OR | 95%CI |
|  |  |  |  |  |  |
| **Sweep**  Age 23  Age 32  Age 42  Age 46  Age 50  (Age 55 = ref.) | **0.72**  **1.52**  **1.44**  **1.27**  0.99 | **(0.63-0.82)**  **(1.36-1.69)**  **(1.31-1.58)**  **(1.15-1.39)**  (0.91-1.08) | **Sweep**  Age 30  Age 34  Age 38  (Age 42 = ref.) | **0.45**  **0.43**  1.06 | **(0.39-0.55)**  **(0.38-0.50)**  (0.97-1.16) |
| **Region** |  |  | **Region** |  |  |
| North West  East and West Riding  North Midlands  Midlands  East  South East  South  South West  Wales  Scotland  (North = ref.) | 1.01  0.96  **1.27**  1.02  1.12  **1.21**  1.22  1.02  1.22  **1.60** | (0.83-1.22)  (0.80-1.19)  **(1.03-1.57)**  (0.83-1.25)  (0.90-1.39)  **(1.01-1.46)**  (0.97-1.54)  (0.81-1.28)  (0.97-1.55)  **(1.31-1.96)** | Yorks and Humberside  East Midlands  East Anglia  South East  South West  West Midlands  North West  Wales  Scotland  (North = ref.) | **0.68**  0.99  1.18  1.03  1.03  0.82  0.88  1.37  1.04 | **(0.51-0.90)**  (0.73-1.37)  (0.82-1.70)  (0.81-1.31)  (0.75-1.40)  (0.62-1.09)  (0.67-1.16)  (0.99-1.88)  (0.78-1.39) |

Bolded estimates are statistically significant at the *p* < .05 level*.*

**SUPPLEMENTARY TABLE 3**

Health and voting in the 1958 and 1970 cohorts: sensitivity analyses

|  |  | Model 1  Full | | Model 2  Full in 2+ sweeps | | Model 3  Full in 3+ sweeps | | Model 4  Full without  IP weight | | Model 5  Full without  intention to vote | |
| --- | --- | --- | --- | --- | --- | --- | --- | --- | --- | --- | --- |
|  |  | OR | 95%CI | OR | 95%CI | OR | 95%CI | OR | 95%CI | OR | 95%CI |
|  |  |  |  |  |  |  |  |  |  |  |  |
| **NCDS 1958** | **Self-rated health**  Fair  Poor or worse  (Good or better = ref.) | **0.85**  0.83 | **(0.77 - 0.94)**  (0.69 - 1.00) | **0.85**  0.84 | **(0.77 - 0.93)**  (0.70 - 1.02) | **0.85**  0.83 | **(0.77 - 0.94)**  (0.68 - 1.00) | **0.86**  **0.83** | **(0.80 - 0.94)**  **(0.69 – 0.99)** | **0.85**  **0.83** | **(0.77 - 0.93)**  **(0.70 – 0.99)** |
|  | **Limitations in everyday activities** |  |  |  |  |  |  |  |  |  |  |
|  | Limited  (No = ref.) | 1.11 | (0.98-1.25) | 1.11 | (0.98-1.25) | 1.13 | (0.99-1.27) | 1.11 | (0.98-1.24) | 1.07 | (0.96-1.20) |
|  |  |  |  |  |  |  |  |  |  |  |  |
| **BCS 1970** | **Self-rated health**  Fair  Poor or worse  (Good or better = ref.) | **0.82**  **0.68** | **(0.72 - 0.95)**  **(0.52 - 0.90)** | **0.83**  **0.70** | **(0.72 - 0.96)**  **(0.53 - 0.92)** | **0.84**  **0.74** | **(0.72 - 0.97)**  **(0.55 - 0.99)** | **0.84**  **0.67** | **(0.73- 0.97)**  **(0.52 - 0.86)** | **0.78**  **0.63** | **(0.68 - 0.90)**  **(0.48 - 0.83)** |
|  | **Limitations in everyday activities** |  |  |  |  |  |  |  |  |  |  |
|  | Limited  (No = ref.) | 1.07 | (0.87 - 1.31) | 1.07 | (0.87 - 1.32) | 1.09 | (0.87 - 1.36) | 1.11 | (0.93 - 1.30) | 1.06 | (0.86 - 1.30) |
|  |  |  |  |  |  |  |  |  |  |  |  |

Estimates represent odds ratios (OR) from weighted and unweighted random-effects logistic models. Bolded estimates are statistically significant at the *p* < .05 level. The full model controlled for: 1) time-invariant: age, sex, region, mother’s age, mother’s smoking, mother’s weight, father’s social class, birth weight, intention to vote in the next election, educational attainment; 2) time-varying: social class, employment status, parenthood, marital status, housing tenure.

OR = Odds ratio. CI = Confidence interval. IP = inverse-probability
